# Potential Benefits from Physical Exercise in Advanced Cancer Patients Undergoing Systemic Therapy? A Narrative Review of the Randomized Clinical Trials

**DOI:** 10.3390/curroncol31120563

**Published:** 2024-12-01

**Authors:** Federico Bozzetti

**Affiliations:** Freelance Surgeon Oncologist, Residenza Querce, Milanodue, 20054 Segrate, Italy; federicobozzetti@gmail.com; Tel.: +39-329-765-5385

**Keywords:** physical exercise in cancer patients, resistance training in cancer patients, aerobic exercise in cancer patients, high-intensity interval training in cancer patients, compliance with physical exercise in cancer patients, home-based versus supervised exercise in cancer patients

## Abstract

Design. The purpose of this review is the analysis of the literature concerning the effects of physical exercise in cancer patients undergoing medical oncologic treatment. Papers were retrieved from the scrutiny of 15 reviews/meta-analyses published in the last 2 years, which, however, pooled different populations of patients (surgical and medical patients, receiving or not an oncologic therapy, harboring a cancer, or being survivors). Results. We reviewed the data of 35 RCTs on the use of physical exercise in cancer patients, distinguishing well-nourished from malnourished patients. The conclusions of our study are the following: No major difference between well-nourished and malnourished patients as regards compliance/adherence with physical exercise and outcomes. Compliance with physical exercise was reported in about 70% of the studies. Compared with a control group receiving the usual care, in patients who practiced physical exercise, a benefit in some parameters of physical function and quality of life and lean body mass (LBM) was reported in 61%, 47%, and 12%, respectively, of the studies in non-malnourished patients, and in 50%, 100%, and 36%, respectively, of the studies in malnourished patients. The benefit in LBM was more frequently reported in weight-losing patients. There was no strict association among the results of different outcomes (muscle function vs. quality of life vs. LBM). There are still some ill-defined issues, including the optimal physical regimen (with some authors favoring high-intensity interval training and resistance) and the place of exercising (patients usually preferring home exercises, which, however, have been proved less efficacious).

## 1. Introduction

Depletion of the muscle mass is prevalent in patients with cancer and is more frequent when cancer is in the upper GI tract, pancreas, lung, head and neck, and in the advanced stages of disease [1,2,3,4,5]. Low muscle mass has been consistently associated with adverse clinical outcomes, such as higher morbidity and mortality, dose-limiting toxicity, poor compliance with oncologic therapy, and length of hospitalization [6,7]. Quality of life (QoL) is also affected following cancer diagnosis and is positively associated with muscle mass [8,9,10,11,12,13].

Physical exercise is a globally recognized way of increasing muscle mass in healthy people as well as in elderly sarcopenic ones; however, its potential role in hypoanabolic procatabolic cancer patients is still poorly appreciated in the scientific oncologic community. The current question is whether physical activity is able to support muscle mass and muscle function in advanced cancer patients undergoing an oncologic therapy and whether this might translate into a clinical benefit.

Although several reviews [14,15,16,17,18,19,20,21,22,23,24,25,26,27,28] dealing with the effects of the physical exercise in patients with advanced cancer have been published in the years 2022–2023, it is still difficult to reach an unambiguous conclusion for a variety of reasons. Some reviews simply pool in their analysis the RCTs, unpublished controlled trials, prospective or retrospective comparative studies, or single-arm studies, most include both tumor-bearing patients and survivors, patients undergoing surgery and receiving an oncologic therapy, patients receiving chemotherapy for an advanced tumor and those receiving adjuvant therapy, hence potentially including tumor-free patients. Finally, most of the studies focused on the feasibility of the physical activity as a primary endpoint of the research, and one systematic review and meta-analysis [26] only focused on adverse events, health-care utilization, and treatment tolerability.

The aim of this review is the analysis of RCTs comparing physical exercise versus usual care in patients with an advanced cancer receiving chemotherapy, with a special focus on change in the fat-free mass (FFM) or in the muscle function and their potential correlation with QoL.

## 2. Methods

The above-mentioned recent reviews [14,15,16,17,18,19,20,21,22,23,24,25,26,27,28] were used to retrieve the pertinent selected literature. In addition, a search of the recent papers published in the first 4 months of 2024 was performed through PUBMED. The inclusion criteria were the same for both the papers retrieved from previous recent reviews and the papers published in the early months of 2024.

We only considered full papers of RCTs in adult cancer patients with an advanced disease, receiving chemotherapy, where the experimental arm underwent a planned program of physical exercises, and the control group was kept under its usual practice. The papers dealing with surgical patients undergoing a preoperative prehabilitation or receiving adjuvant chemotherapy after a successful curative surgery (who can virtually be considered tumor-free) or practicing an exclusive selective physical therapy aimed to potentiate the ventilatory function or the pelvic muscle function in lung cancer or urogenital tumor patients were not considered. Both abstracts and papers not published in English were excluded. Finally, the papers were divided into two large series depending on the baseline nutritional status. Patients were considered malnourished if they had lost weight, or the BMI was below 19.5, or NRS2002 was >3, or the ECOG score was ≥3, or they were receiving tube feeding. The comparison of the effects of physical exercise was always inter-group (and not intra-group), hence the symbol “↑” does not necessarily mean an absolute benefit but also a lower deterioration inthe experimental group as compared with the control group.

## 3. Results

We collected 35 RCTs (1874 patients) [29,30,31,32,33,34,35,36,37,38,39,40,41,42,43,44,45,46,47,48,49,50,51,52,53,54,55,56,57,58,59,60,61,62,63,64,65] and divided them into two groups, one dealing with non-malnourished patients (25 RCTs, 1266 patients) (Table 1 and Table 2), and the other with malnourished patients (10 RCTs, 608 patients) (Table 1 and Table 2).

On average, exercise programs had a mean of 3.5 sessions per week, each aerobic session usually included 10–15 min of warm-up, followed by 30 min of training with a wide range of heart rate goals, while in contrast, resistance training often required 10–20 min. Aerobic training aimed at improving VO2 max (or its surrogates), while the goal of resistance training was to increase the muscle mass. Common resistance training included resistance bands and free weights, where participants could perform sets of repetitions in one or more muscle groups with the goal of achieving a pre-specified rating of perceived exertion [30], as described by Borg et al. [66].

Most experiences were in non-malnourished cancer patients and the data of 25 RCTs are reported in Table 1 and Table 2. Lung and GI accounted for the most prevalent primary cancers and median and mean compliance with the planned program of physical exercise were 70% and 70% (r. 37–100), respectively. Most primary endpoints referred to muscle function/physical performance or QoL parameters but at least eight focused on feasibility [35,38,45,46,48,49,51,53]. Median and mean age of patients were 63 and 57.6 (r. 32–66) years, respectively. Patients performed physical exercises for a median period of 3 months, mean 9.3 (r. 2.2–24) weeks. Out of 25 studies, 18 reported the effect of the physical exercises on muscle function and 11 (61.1%) [29,31,32,33,35,42,45,46,47,52,58] showed some benefit, and in 4 of them (36.3%) [31,32,33,52,60] patients also improved their QoL. However, QoL improved also in two studies [30,43] despite no benefit of muscle function. Globally, QoL was evaluated in 19 studies and some items of QoL improved in 9 of them (47.3%). Lean body mass (LBM) change was reported in eight studies and only in one (12.5%) [65] was improved. Finally, four studies evaluated both LBM and QoL: there were no benefits in LBM but only one benefit [65] in QoL.

Ten studies included malnourished patients (Table 1 and Table 2) and the vast majority were patients with GI tumors, especially pancreatic. Four of them focused on feasibility [40,41,56,61] and median and mean compliance were reported in 69% and 68% (r. 59–90) of patients, respectively. The median and mean age of the patients were 61 and 63.2 (r. 57–78) years. Patients performed physical exercises for a median and mean period of 10 weeks and 13 weeks (r. 4–24), respectively. Six studies evaluated the muscle function, and a benefit was observed in three (50%) [44,50,63] and all these three (100%) reported an improvement of QoL. QoL was improved in three [37,57,61] out eight studies (37.5%) and, similarly, LBM changes were reported in eight studies and improved in three (37.5%) [44,50,63] concurrently with a benefit in muscle function. It is noteworthy, however, QoL improved also in one study [37] which did not exhibit any benefit in muscle function.

We attempted to explore whether the number of weeks of exercise and dietary supplementation were related with some outcomes and the findings are reported in Table 3 and Table 4.

However, we were not able to find any data indicating that the duration of the exercise as well as administration of a nutritional supplementation could affect the results. Especially as regards the duration of the exercise, both positive and negative results were achieved in quite similar time frames. The different methodology of exercise and the impact of different regimens of chemotherapy can account for the difficulty of assessing the more advantageous approach.

## 4. Discussion

The aim of this review was to define the potential of physical exercise in advanced cancer patients on chemotherapy and mainly relies on the RCTs quoted in the systematic reviews and meta-analyses published in the last two years. Although several scientific societies [66,67,68,69,70] recommend engaging in at least 90 min of moderate-intensity aerobic physical activity per week, in addition to muscle strengthening activities twice weekly, exercise is only performed regularly by a minority of patients diagnosed with cancer [71,72].The conclusion of a recent Cochrane review [73] including only four studies, stating that it was not known if exercise is helpful or safe for people with cancer who experience loss of appetite and weight loss, may have argued, among the oncologists, against a wider use of physical activity.

Our analysis shows that compliance with the physical exercise was about 68–70%, even if estimates of physical activity based on self-report are generally higher than estimates derived from objective measures [74,75,76] and, hence, self-reported adherence must be interpreted with caution. Our findings agree with other meta-analyses [77,78] that show a low incidence of adverse events from participation in exercise oncology clinical trials during treatment and that engaging in exercise during treatment through aerobic exercise interventions and strength training is usually well tolerated without adverse events.

Since we hypothesized that feasibility, compliance, and possibly some outcomes might be different in patients malnourished or not, we separately examined these two series of studies. However, data show that adherence to the program of physical exercise was almost identical, 70% versus 68–69% in non-malnourished and malnourished patients, respectively. There was some benefit as regards the muscle function in 61% versus 50%, the QoL in 47.3% versus 100%, and LBM in 12.5% versus 37.5% of studies on non-malnourished and malnourished patients, respectively. The better response of the physical exercise on LBM in malnourished patients might be due to the combination of the physical exercises with a supervised nutritional support in six of ten of these studies, might simply reflect a major hindrance in increasing LBM in subjects who do not have a baseline muscle mass depletion.

Due to the impossibility of considering all variables (type of tumor, oncologic treatment and response, duration, and characteristics of the exercise regimen etc.) affecting the response to the exercise in the different studies, a formal statistical analysis was not performed; however, it appears that the duration and combination with a nutritional supplement did not affect the outcome (Table 3 and Table 4).

The most intriguing observation was the frequent dissociation of the response of the different endpoints. For instance, in one study [50], muscle function and LMB improved but QoL did not, and on the contrary, QoL improved in a study [37] without any positive response of the muscle function. However, in three studies [44,50,63] where LBM improved, there was a concurrent benefit in muscle function. Overall, these data should warn the clinicians to not consider muscle function and LBM as a surrogate of QoL.

The analysis of the literature shows that there are two major issues which remain unresolved, and these certainly represent a limitation of our analysis.

The first concerns the option of home versus supervised exercise. Comparing with home exercise programs, supervised sessions appear to generate higher adherence and social support and improve both role and emotional functioning [79], likely through interactions with supervising personnel and peers in the exercise setting. This would also provide a number of additional benefits to participants with a poor prognosis and symptomatology [80,81]. Kuehr et al. [82] showed a 95% adherence rate in a hospital-based exercise program and 77% adherence rate in a home-based program, while Quist et al. [83] reported a rate of 73.3% and 8.7% in supervised group training and home-based training, respectively. Consequently, supervised exercise programs (especially resistance training) appear more effective than non-supervised ones [44,61,84]. Notably, most patients prefer home-based exercises [85], even if accessibility of the location would appear to be more important than the type of location [51,86]. Since at-home exercise programs may be related to self-efficacy at baseline, they might be a better option for patients with high self-efficacy and motivation [87]. To improve the adherence to the exercises the use of step trackers or of accelerometers or recalls by phone have been proposed [88].

The second issue which warrants further deep exploration in future studies is the modality of the exercises. Most of the RCTs reported in this review, as well as the guidelines by American College of Sports Medicine, propose a combination of both resistance and endurance training in cancer patients, since concurrent training can induce wide-ranging physical adaptations promoting both aerobic (VO2 max and resistance to fatigue) and anaerobic (muscle strength and function) components simultaneously, without any adverse effect in cancer patients. This is in keeping with both the guidelines of the American College of Sports Medicine as well as with other studies not considered in this report [89,90,91,92]. However, some patients with advanced gastrointestinal cancer [93] would prefer resistance exercise training since resistance exercise training has a higher potential for individualization. While general recommendations include primarily aerobic exercise of moderate intensity, the definitive superiority of one exercise modality over another is still uncertain, especially in advanced-stage lung cancer patients [67]. This review shows that several regimens of physical exercise were applied in the different studies because the optimal training program is still ill-defined. Reljic et al., [53] following the observation that the fatigue was a common reason for the participant reporting difficulty performing their prehabilitation schedule, favored the incorporation of low-high-intensity interval training into exercise programs and/or to implement it as an initial preparatory training modality before higher-volume exercise regimes. Toohey et al. [94] showed that low-volume high-intensity training was associated with greater improvements in cardio-respiratory fitness, lower body strength and waist circumference compared with traditional continuous low- to moderate-intensity exercise training, even if in a previous study, the same authors [95] reported that the low-volume high-intensity program was well tolerated by the participants and hence was probably the preferred modality to improve fitness. Three recent systematic reviews and meta-analyses [95,96,97] reported that high-intensity interval training was superior compared to usual care in improving physical fitness, peak oxygen consumption, and health-related outcomes across all stages of therapy and aftercare. Currently, there is no evidence for the benefits of high-intensity interval training compared to aerobic training of moderate intensity for changes in cardiorespiratory fitness, LBM, and patient-reported outcomes [21]. Recent studies [98,99,100] have shown no significant differences between high-intensity interval training and control groups in terms of body composition, but high-intensity interval training prevented body weight increase in breast cancer patients compared to the usual care control group. More recently, the use of whole-body electro-myostimulation has been proposed as a way of potentiating the biomechanical function of muscle [101].

Further limitations of this paper concern the heterogeneity of patients included in different RCTs as well as in the same study, which precludes a more precise analysis by type of primary.

However, there are some points of force, as follows: a good compliance and adherence to the physical exercise program was commonly reported, some primary endpoints were achieved (muscle function and QoL in half or more of the studies and LBM in a lower rate), and the presence of malnutrition was not a major hindrance to the programs as regards both the compliance/adherence and the outcomes. Additional randomized and controlled studies are needed to determine the optimal intensity and quantity of training programs to achieve possible positive effects. More important, patients and their formal and informal caregivers should be alerted that exercise is safe and feasible, can be tailored to individual needs and levels of functioning, and can be performed both at home without supervision and in a supervised setting. Finally, an important topic which was beyond the scope of this review but is of utmost importance, is the possibility to achieve, through the physical exercise, a better response to the oncologic treatment as recently shown by randomized [102] and non-randomized studies [65].

## Figures and Tables

**Table 1:** (**a**) Details of the RCTs in non-malnourished cancer patients. (**b**) Details of the RCTs in malnourished cancer patients.

(**a**)						
**Author**	**Primary** **Endpoint**	**Patient ** **Population**	**Ns/Nrs/Ps**	**W** **K**	**Exercise** **Intervention**	**Measured Outcomes**
Baumann [32] (2010)	ET, strength, QoL	N 64; m/f 1.3; 44 yrs.; pts undergoing HSCT	BMI 24; PS 90	3	AT 20 min/d	EORTC QLQ-C30, ET watts and time
Baumann [33] (2011)	ET, strength, lung function, QoL	N 33; m/f 0.9; 32 yrs.; leukemia/lymphoid tumors	BMI 24	2.2	ET 20–30 min/d., Borg intensity ‘slightly strenuous’/‘strenuous’.	Strength Digimax2000—load cell; forced vital capacity; EORTC-QLQ C30
Hwang [43] (2012)	Exercise capacity	N 24; m/f 1; 60 yrs.; lung	BMI 22	8	HIIT (80% VO2 peak, ≥3 session/wk. and RE ≥2 sessions/wk. (30–40 min/d)	VO2 peak, EORTC QLQC30, muscle strength, endurance
Hornsby [42] (2014)	Safety, efficacy	N 20; f 49 yrs.; breast	BMI 29	12	AT (60–100% of VO2 peak) 3 sessions/wk.	CPET, FACT-B
Alibhai [29] (2015)	QoL, fatigue, fitness	N 81; m/f 1.2; 58 yrs.; leukemia	BMI 27; ECOG 0–1	5	mod-intensity (5 d/wk., 30–60 min/session)	Global QoL, FACT-F, 6MWT distance
Capozzi [34] * (2016)	Optimal timing for initiation of the 12-wk. intervention	N 60; m/f 4.4; 56 yrs.; head neck	BMI 27; LBM 57 kg PG-SGA 6.3	12–24	short, mod intensity warm-up followed by 2 sets of 8 reps, 8 to 10 RM for 10 exercises	HGST, 6 min walk, 30 s to stand, sit, and reach, flexibility, FAACT, FENSI-22, DEXA
Dhillon [36] (2017)	Fatigue	N 111; m/f 1,2; 64 yrs. lung	BMI 26,2; ECOG ≤2	8	Sessions ≈1 h:45 min PA; 15 min behavior support. PA was predominantly aerobic.	FACT-F, EORTC-QLQ-C30, physical or functional status scores
Uster [59] (2017)	Physical performance, NS, QoL.	N 58; m/f 2.2; 63 yrs.; lung and GI	BMI 25.8; BW 72.3 kg.; PS ≤ 2; QoL 61.6 NRS ≤ 2 in 70% pts	12	60 min session, 2/wk.; strength training (60–80% 1-RM) in 2 sets of 10 rep.; balance training	EORTC QoLQ-3.0, PS, HGST, 6 min walk test, TSTS, 1-RM leg press, 3 d dietary record, BW, BIA
Vanderbyl [60] (2017)	Anxiety, depression, QoL.	N 24; m/f 1.4; 64 yrs. GI, lung	PS 0–1	12	45 min walking sessions vs. ET and strength at 60–70% max heart rate or 2–4 METs.	HADS score, Fact-G, 6MWT
Zhang [64] (2017)	Fatigue	N 91; m/f 3, 63 yrs., lung	ECOG most 0–1	12	Tai-Chi exercise	MFSI-SF
Christensen [35] (2019)	Safety and feasibility	N 50, m/f 9; 65 yrs.; esophagus	BMI 28	9	75 min HIIT, 2/wk.	HGST, BIA, CT scan, DEXA, FACT-E
Egegaard [38] (2019)	Feasibility	N 15; m/f 2; 64 yrs.; lung	BMI 24	7	20 min mod/HIIT aerobic ×5/wk.	VO2 peak, 6MWD, FEV1, FACT-L, HADS
Lee [45,46] (2019/2020)	Feasibility	N 30, 47 yrs., breast	BMI 32	8	20 min HIIT, 3/wk.	VO2 max, FACT-B, MFI-20, FFMQ-15, TUG, TSTS), SCT, 6MWT
Rutkowska [55] (2019)	Impact of exercise training	N 30; m/f 9; 60 yrs.; lung	BMI 25; ECOG 1–2	4	cycle ergometer (20–30 min at 30–80% of peak work rate), treadmill (40–70% of the 1-RM, Nordic walking (45 min)	Up and go, chair stand, arm curl
Moug [48,49] (2019/2020)	Feasibility	N 48; m/f 2.6; 66 yrs.; rectum	BMI 20–30 in 77% pts	14	daily step count by 3000 above their baseline value	Step count, BDI-II, FACT-C, PANAS, EORTC-QLQ CR29/C30
Quist [52] (2020)	VO2 peak	N 218; m/f 0.9; 64 yrs.; lung	BMI 24; ECOG 0–1 in 90% pts	12	2/wk. warm-up (10 min, 60–80% HRmax), strength training (3 sets of 5–8 rep. 70–90% of 1 RM)	VO2 peak, muscle function, 6 min walk
Storck [58] (2020)	Physical function	N 52; m/f 1.3; 63 yrs.; various tumors	BMI 25.8; PS 1–2; NRS 1–2 in >80%	12	AT, RE, coordination training, intensity Borg 4–6, ×3/wk.	EORTC QLQ-C30, BFI, HGST, 60 s sit-to-stand, timed up and go, SPPB
Wochner [62] (2020)	Muscle and adipose tissue compartments	N 53; m/f1.6; 62 yrs.; pancreas	BMI 23.9	24	8 sessions (2–3 sets/8–12 rep (14–16 Borg scale)	CT scan, isokinetic dynamometer
Bade [31] (2021)	PA, QoL, depression Scores, biomarkers	N 40; m/f 3; 65 yrs.; lung	ECOG 0–1	12	<150 min/wk. of mod. or 75 min/wk. of vig. intensity PA	Fitbit^®^ Flex2, EORTCQLQ-C30; MAQ
Rutkowska [54] (2021)	QoL	N 26; m/f 13; 61 yrs.; lung	BMI 23; ECOG 0–1	2	30 min on cycle ergometer/treadmill at 30% to 80% of peak work, weighted exercise eat 40% to 70% of the 1-RM),Nordic walking (45 min)	SF-36, FACT-L
Allen [30] (2022)	Anaerobic threshold at cardiopulmonary exercise testing	N 54; m/f 5.7; 64 yrs.; esophagogastric	BMI 27.9	15	AT (2 sessions/wk.; 60 min/d; mod intensity), RE ×3/wk.; 60 min/d; mod intensity)	VO2 peak, HGST, skeletal muscle mass, EORTC QLQ-C30
Piraux [51] (2022)	Feasibility	N 18; m/f 2.6; 62 yrs.; rectum	BMI 23	5	HIIT ≥ 85% of max HR (220—age) vs. RE (1–3 sets of 8–12 rep of 8 ex at 4–6 (mod. Borg scale) vs. UC	fatigue, health-related QoL
Reljic [53] (2022)	Feasibility, safety, efficacy	N 24, m/f 1.1; 55 yrs., various	BMI 24; KI 73	12	HIIT (80–95% HR peak): 2/wk. for 12 wk., vs. light mobilization ex	FACIT, EORTC QLQ-C30, KI
Zylstra [65] (2022)	Chemotherapy response	N 42: m/f 6; 65 yrs.; esophagus	BMI 26	8	combined AT and strength training, mod intensity (4–5 of a 0–10 perceived exertion scale)	CT scan
Li [47] (2024)	Functional ability and nutritional status	N 121; m/f 2.1; 86% < 65 yrs; stomach	21% BMI ≥ 24	7–9	AT 30 min moderate intensity (12–13 of Borg score ranging 6 to 20) × 3 d/wk.; RE 3 sets of 8–12 rep for 8 muscle groups (5–6 on a 10-point scale)	6MWD
(**b**)						
**Author**	**Primary** **Endpoint**	**Patient ** **Population**	**NS/NRS/PS**	**Wk**	**Exercise** **Intervention**	**Outcome Measures**
Forget [39] (2014)	Cachexia management	N 54; various T; pts at high risk for cachexia	BMI 24; WL ≥ 5% in the prior 6 mos.; PS: 0–2	12	Daily physical exercise	BW; HGST; MAMC; QLQ C-30; PG-SGA
Xu [63] (2015)	Walking capacity, NS	N 56, m/f 7; 60 yrs. esophagus	dysphagic	4–5	3 times/wk., 20 min, at maximal HR formula: ([220-age] × desired intensity of 60%).	6MWT, HGST, BIA, BW
Solheim [56] (2017)	Feasibility, safety	N 46; m/f 1.5; 61 yrs.; lung, pancreas	WL 5.6% in 6 mos.	6	Combined home-based AT and RE	L3 CT scan, HGST, 6MWT, PG-SGA, FSS
Grote [40] (2018)	Feasibility	N 20; m/f 3; 61 yrs.; head neck	WL 7.1%	8	3 (30 min) sessions/wk., 8–12 rep. last set at submaximal one-rep	BIA, MFI; FAACT; 6MWT
Edbrooke [37] (2019)	Change in functional exercise capacity (6MWD)	N 92; m/f 1.2; 64 yrs.; lung	Cachexia 36%, ± frailty 90%	8	AT (at 4 of Borg Scale), RE (8–10 rep., 2–3 sets, mod intensity), 2–3 sessions/wk.	BREQ-2; FACT-L; HRQoL; PA; PAAI.
Wiskemann [61] (2019)	Feasibility, effectivity on muscle strength	N 25; m/f 1.2; 67 yrs.; pancreas	BMI 23.4, 44.4% pts with WL ≥ 10% in 6 mos.	24	At week 5, ex increased till 8 per session (60 min): 3 sets with 8–12 rep (mod-vig intensity (60–80% 1-RM).	Isokinetic and handheld dynamometer, CPET, 6MWD
Steindorf [57] (2019)	Physical function at 6 mos.	N 47; m/f 1.2; 60 yrs.; pancreas	BMI 23.7; 53.1% pts with WL ≥ 10% in 6 mos.	24	Till 8 ex/session 2–3 sets with 8–12 rep. (60–80% 1-RM), 14–16 (Borg Scale)	EORTC QLQ-C30, EORTC-PAN26, MFI
Kamel [44] (2020)	Mobility, muscle strength, LBM	N 40; m/f 1.8; 52 yrs.; pancreas	BMI 21.1; WL > 5% in 6 mos. or >2% if BMI < 20	12	At wk. 5, 8 ex/session (3 sets with 8 to 12 rep and mod-high frequency (60–80% 1-RM).	Chair rise time: 400-m walk performance; maximum isokinetic peak torque; DEXA
Hall [41] (2021)	Feasibility of exercise, nutrition program	N 45; m/f 1.4; 78 yrs.; various tumors	BMI 26; WL 5% in 6 mos.	8	AT and RE; 60 min ex/wk., mod intensity (Borg scale 3–4)	2-min walk test, timed up and go, daily step count Life space assessment, EORTC QLQ-C15-PAL, BW
Ngo-Huang [50] (2023)	Physical function	N 151; m/f 1.5; 66 yrs.; pancreas	NRS 7; sarcopenia 51%	22	≥30 min mod AT ≥ 3/wk. plus ≥2 RE/wk.	6MWD, 5× tSTS, arm curl test, HGST, GSLTPAQ, PROMIS, FACT-Hep, CT scan

AT: aerobic training; BDI-II: Becks Depression Inventory; BIA: bioelectrical impedance analysis; BIF: brief fatigue inventory; BW: body weight; CFR: cancer-related fatigue; CPET: cardiopulmonary exercise testing; d:day; DEXA: dual-energy X-ray absorptiometry; EORTC-PAN26: pancreas-specific module; ET: endurance training; ex: exercise; FAACT: Functional Assessment of Anorexia/Cachexia Therapy; FACT-B: functional assessment of cancer therapy–breast; FACT-E: functional assessment of cancer therapy–esophagus; FACT-Hep: functional assessment of cancer therapy–hepatobiliary; FACT-L: functional assessment of cancer therapy–lung; FEV1: pulmonary function; FFMQ-15: Five-Facet Mindfulness Questionnaire; FSS: fatigue severity scale; HADS: anxiety and depression; HGST: hand grip strength; HIIT: high intensity interval training; HR: heart rate; KI: Karnofsky index; LBM: lean body mass; MAMC: mid-upper muscle circumference; MFI: Multidimensional Fatigue Inventory; mos.: months; med: median; MAQ: Modifiable Activity Questionnaire; Mod.: moderate; NRS: nutritional risk score 2002; NS: nutritional status; PA: physical activity; PANAS: Positive and Negative Affect Schedule; PG-SGA: patient-generated Subjective Global Assessment; PMW: predicted maximal workload; PS: performance status; QoL: quality of life; RE: resistance exercise; Rep: repetitions; RM: maximum repetition; rep: repetitions; SCT: stair climb test; SMD; skeletal muscle density; SMI: skeletal muscle index; SPPB: short physical performance battery; T: tumors; TUG: timed up and go; TSTS: timed sit-to-stand test; UC: usual care; Vig: vigorous; WK.: weeks; WL: weight loss; VO2 peak: peak oxygen consumption; 6MWT: 6-Minute Walk Test. * Patients randomized for exercise intervention during or after radiation therapy. BREQ-2: Behavioral Regulation of Exercise Questionnaire; ∆: statistical difference; GSLTPAQ: Godin–Shephard Leisure-Time Physical Activity Questionnaire; HRQoL: functional assessment of cancer therapy–lung; one RM: one-repetition maximum; PAAI: Physical Activity Assessment Inventory; PROMIS: patient-reported Outcomes Measurement Information System.

**Table 2:** (**a**) Results of the RCTs in non-malnourished cancer patients. (**b**) Results of the RCTS in malnourished cancer patients.

(**a**)							
**Author**	**Oncol Therapy**	**Nutr.** **Supp**	**Muscle Function**	**QoL**		**Nutr** **Status**	**LBM**
Baumann [32] (2010)	CT	nr	↑ ET	↑ Global QoL, physical functioning		nr	nr
Baumann [33] (2011)	CT	nr	↑ ET	QoL no ∆		BW no ∆	nr
Hwang [43] (2012)	TA	nr	No ∆	↑ fatigue		nr	nr
Hornsby [42] (2014)	CT	nr	↑ VO2 peak	FACT-B no ∆		nr	nr
Alibhai [29] (2015)	Induction CT	nr	↑ MS, time chair stand	QoL, fatigue no ∆		nr	nr
Capozzi [34] (2016)	RT ± surgery ± CT	nr	nr	fitness, QoL no ∆		PG-SGA no ∆	No ∆
Dhillon [36] (2017)	CT or TA	Both arms	No ∆	No ∆		No ∆	nr
Uster [59] (2017)	yes	9–10 g prot, 150–200 kcal 2/wk.	No ∆	QoL no ∆		Nutritional status no ∆	PA no ∆
Vanderbyl [60] (2017)	CT	nr	↑ ET, 6MWT	↑ weakness		nr	nr
Zhang [64] (2017)	CT	nr	nr	↑ MFSI-SF general, physical, vigor scores		nr	nr
Christensen [35] (2019)	CT neo-adj.	nr	↑ peak power, ↑ muscle strength	nr		nr	No ∆
Egegaard [38] (2019)	CT	nr	No ∆	No ∆		nr	nr
Lee (46,47) (2019/2020)	CT	nr	↑ VO2 max, SCT, 6MWT	No ∆		nr	nr
Rutkowska [55] (2019)	CT	nr	↑ up and go	nr		nr	nr
Moug [48,49] (2019/2020)	RT, CT neo-adj.	nr	No ∆	No ∆		nr	No ∆
Quist [52] (2020)	CT	nr	muscle function	↑ FACT social well-being, anxiety, depression		nr	nr
Storck [58] (2020)	CT	Leucine suppl. Prot 1.2 g/kg	↑ HGST	fatigue no ∆		No ∆	No ∆
Wochner [62] (2020)	CT ± surgery	nr	nr	nr		B compartment no ∆	No ∆
Bade [31] (2021)	CT, IM	nr	↑ PAc	↑ EORTC functional domain		nr	nr
Rutkowska [54] (2021)	CT	nr	nr	↑ physical well-being		nr	nr
Allen [30] (2022)	CT neo-adj.	yes	No ∆	↑ EORTC QLQ-C30		nr	nr
Piraux [51] (2022)	CT RT neo-adj.	nr	nr	↑ RES vs. HIIT		No ∆	nr
Reljic [53] (2022)	anticancer treatments	25–30 kcal and >1.0 g prot/kg/d	nr	nr		no ∆	no ∆
Zylstra [65] (2022)	CT neo-adj.	nr	nr	nr		nr	↑
Li [47] (2024)	CT neo-adj.	whey protein (1.2–1.5 g/kg ideal BW) and ONS (400–900 Kcal)	↑ 6MWD	nr		nr	nr
(**b**)							
Forget [39] (2014)	RT + CT	mirtazapine 30 mg/d	weekly dietician advice	nr	HGST, EORTC QLQ-30, no ∆	PG-SGA, BMI no ∆	No ∆
Xu [63] (2015)	RT + CT	nr	weekly dietician advice	↑ walk distance, HGS	nr	↑ BW	↑
Solheim [56] (2017)	PolyCT	Celecoxib 300 mg/d, EPA 2 g/d	ONS: 559 Kcal, 29 g AA/d	No ∆	Fatigue no ∆	PG-SGA, BW no ∆	No ∆
Grote [40] (2018)	RT ± CT	nr	TF 55% of pts	nr	Fatigue no ∆	nr	No ∆
Edbrooke [37] (2019)	CT, RT, TT	nr	nr	No ∆	↑ HRQoL symptom level	nr	nr
Wiskemann [61] (2019)	CT	nr	nr	nr	↑ Physical function	BW no ∆	No ∆
Steindorf [57] (2019)	CT adj/neoadj.	nr	nr	nr	↑ QoL, ↑ Physical function	nr	nr
Kamel [44] (2020)	CT	nr	nr	↑ mobility: ↑ 400 m walk; performance; ↑ chair rise; ↑ MS; ↑ peak torque	nr	nr	↑
Hall [41] (2021)	Incurable pts	nr	660 kcal, 29 g prot./day	step count, Timed up and go test, 2 min walk test no ∆	aPG-SGA score, AveS score no ∆	BW no ∆	nr
Ngo-Huang [50] (2023)	Neoadj. ther.	nr	15–25 g prot. within 1 h after strengthening ex.	↑ strength training	FACT-Hep, PROMIS no ∆	nr	↑

↑: increase; ACT: anticachectic drugs; B: body; BW: body weight; CT: chemotherapy; CT adj.: chemotherapy adjuvant; CT neoadj.: chemotherapy neoadjuvant; ∆: difference; d: day; ET: endurance training; HGS: handgrip strength; HIIT: high intensity interval training; IM: immunotherapy; LBM: lean body mass; MS: muscle strength; nr: not reported; nsa: no statistical analysis; NUTR STATUS: nutritional status; NUTR SUPP: nutritional support; PA: phase angle; Pac: physical activity; PG-SGA: Patient-Generated-Subjective Global Assessment (0–≥9); prot: protein; QoL: quality of life; RES: resistance training; RT: radiotherapy; SCT: stair climb test; TA: targeted agents; TF: tube feeding; VO2 peak: peak oxygen consumption; 6MWT: 6-Minute Walk Test; HGST: handgrip strength test; HRQoL: health-related quality of life; TT: targeted therapy.

**Table 3:** Outcomes by weeks of exercise.

Outcome	Weeks, *n*
Muscle function ↑	8.9
no ∆	9.8
Quality of life ↑	11.6
no ∆	10.1
Lean body mass ↑	10.2
no ∆	11.6

↑: positive response; ∆: difference.

**Table 4:** Dietary supplementation and outcomes.

Supplementation	Muscle Function	Quality Of Life	Lean Body Mass
yes	↑ 2/4 (50%)	↑ 0/5 (0)	↑ 0/3 (0)
no	no ∆ 2/4 (50%)	↑ 3/3 (100%)	↑ 1/5 (20%)

↑: positive response; ∆: difference.
